# Emerging phylogenetic structure of the SARS-CoV-2 pandemic

**DOI:** 10.1093/ve/veaa082

**Published:** 2020-11-10

**Authors:** Nicholas M Fountain-Jones, Raima Carol Appaw, Scott Carver, Xavier Didelot, Erik Volz, Michael Charleston

**Affiliations:** School of Natural Sciences, University of Tasmania, Hobart, 7001, Australia; School of Natural Sciences, University of Tasmania, Hobart, 7001, Australia; School of Natural Sciences, University of Tasmania, Hobart, 7001, Australia; School of Life Sciences and Department of Statistics, University of Warwick, Coventry CV47AL, UK; Department of Infectious Disease Epidemiology, MRC Centre for Global Infectious Disease Analysis, Imperial College London, London W2 1PG, UK; School of Natural Sciences, University of Tasmania, Hobart, 7001, Australia

**Keywords:** COVID-19, virus demography, phylodynamics, spread

## Abstract

Since spilling over into humans, SARS-CoV-2 has rapidly spread across the globe, accumulating significant genetic diversity. The structure of this genetic diversity and whether it reveals epidemiological insights are fundamental questions for understanding the evolutionary trajectory of this virus. Here, we use a recently developed phylodynamic approach to uncover phylogenetic structures underlying the SARS-CoV-2 pandemic. We find support for three SARS-CoV-2 lineages co-circulating, each with significantly different demographic dynamics concordant with known epidemiological factors. For example, Lineage C emerged in Europe with a high growth rate in late February, just prior to the exponential increase in cases in several European countries. Non-synonymous mutations that characterize Lineage C occur in functionally important gene regions responsible for viral replication and cell entry. Even though Lineages A and B had distinct demographic patterns, they were much more difficult to distinguish. Continuous application of phylogenetic approaches to track the evolutionary epidemiology of SARS-CoV-2 lineages will be increasingly important to validate the efficacy of control efforts and monitor significant evolutionary events in the future.

## 1. Introduction

The rapid spread of the novel coronavirus SARS-CoV-2 since December 2019 represents an unparalleled global health threat (Andersen et al. 2020). Within 4 months of emerging from Wuhan in Central China, SARS-CoV-2 has now spread to nearly every country and is a major source of mortality (World Health Organization 2020). The first cases of the virus outside China occurred in Thailand on January 13, and by January 30, there were 83 cases in 18 countries. As of May 19, there were over 4.5 million cases in 203 countries or territories (World Health Organization 2020). Coronaviruses (order: Nidovirales, family: Coronaviridae) are enveloped positive-sense non-segmented RNA viruses that infect a variety of mammals and birds. SARS-CoV-2 is the seventh coronavirus to be identified infecting humans. The closest relatives (RaTG13 and RmYN02, 96% and 93% nucleotide identity respectively) derive from the Intermediate Horseshoe bat (*Rhinolophus affinis*) and the Malayan Horseshoe bat (*Rhinolophus malayanus*) (Zhou et al. 2020), although the original host is yet to be conclusively identified (Andersen et al. 2020). Since spilling over to humans, the virus has diverged rapidly, but it is unclear whether these mutations have resulted in SARS-CoV-2 lineages with different epidemiological and evolutionary characteristics (Eden et al. 2020; Korber et al. 2020; Pachetti et al. 2020; Rambaut et al. 2020; Tang et al. 2020; van Dorp et al. 2020). Several lineages have been highlighted for potential significance (Eden et al. 2020; Korber et al. 2020; Tang et al. 2020; van Dorp et al. 2020). For consistency, we adopt the nomenclature outlined by Rambaut et al. (2020), which classifies the initial Lineages as A and B labelled ‘S’ and ‘L’ (in the GISAID nomenclature, Tang et al. 2020). There is some evidence that Lineage A is ancestral to the more recent Lineages B (Rambaut et al. 2020), even though the earliest assembled genomes from December 2019 belong to lineage B (Rambaut et al. 2020; Tang et al. 2020). Sequences within Lineage A and the closest known bat virus share two nucleotides in ORF1ab and ORF8 genes that are not found in Lineage B (Rambaut et al. 2020). More recently, a new Lineage ‘G’ (in the GISAID nomenclature) has been documented originating in Europe in February (Korber et al. 2020). For consistency, we call this Lineage C. It is currently unclear if these lineages differ phenotypically, or whether these lineages show distinctive demographic signatures (i.e. diversity increasing, plateauing, or declining). Any further population sub-structure within these three lineages is also unknown at this point.

Pathogen population structure and effective population size can provide key insights into the epidemiology of an outbreak, such as whether intervention strategies are working to contain spread (i.e. is effective population size declining, Dellicour et al. 2018). Population structure may also align with geography, reflecting the contact structure of the host population. Understanding these variations is important both for vaccine development and evaluating the impact of control efforts across the globe. Detecting structure, particularly in recently emerged outbreaks, is a challenge as these patterns within the data can be cryptic (Volz et al. 2020). For example, some lineages within a population can be rapidly expanding whereas others can be stationary (Volz et al. 2020). Utilizing large numbers of sequences provided by GISAID (Elbe and Buckland-Merrett 2017) and recently developed phylodynamic tools, we interrogate SARS-CoV-2 population patterns to identify ‘hidden’ structure in the pandemic and investigate whether lineages are geographically partitioned and/or are on distinct demographic trajectories.

### 1.1 Three distinct lineages

**Figure 1.**
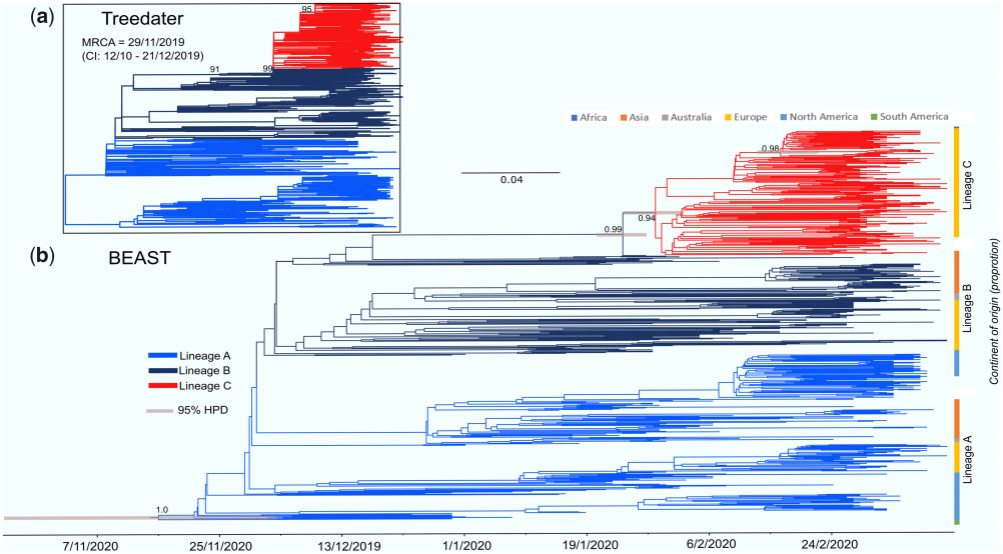
*Treedater* maximum likelihood tree (a) and Bayesian time-scale phylogeny (b) revealing the three SARS-CoV-2 lineages we identified with unique demographic signatures (Lineages A, B, and C). Branches in both trees are coloured by lineage (see Section 2 for details). Most recent common ancestor estimates from the *treedater* analysis are also provided. Density bars are shown representing the 95 per cent highest posterior density (HPD) intervals for the dating of each lineage. Node posterior support values and bootstrap support values are shown for internal nodes not leading to leaves with values >0.8 or 80 percent posterior or bootstrap support, respectively. See Supplementary Fig. S1 for the Bayesian tree with all posterior support values. Stacked bar plots show the proportion of sequences from each country classified in each lineage.

Our analyses show support for three distinct lineages of SARS-CoV-2 actively spreading around the world (Fig. 1). These lineages are highly unlikely to have been generated under the same coalescent process (*P *<* *0.0001 for each pairwise *treestructure* test, see Section 2) and the same analysis performed on our maximum clade credibility (MCC) Bayesian phylogeny yielded very similar results (Fig. 1). However, *treestructure* tests on a sample of Bayesian posterior trees revealed that this result was sensitive to phylogenetic uncertainty with, for example, one lineage (Lineage B, see below) only distinguishable in some of the posterior trees (see Supplementary Table S1). Nonetheless, given the balance of evidence presented here and in previous work (Eden et al. 2020; Tang et al. 2020), Lineage B is likely distinctive from Lineage A and Lineage C.

**Figure 2.**
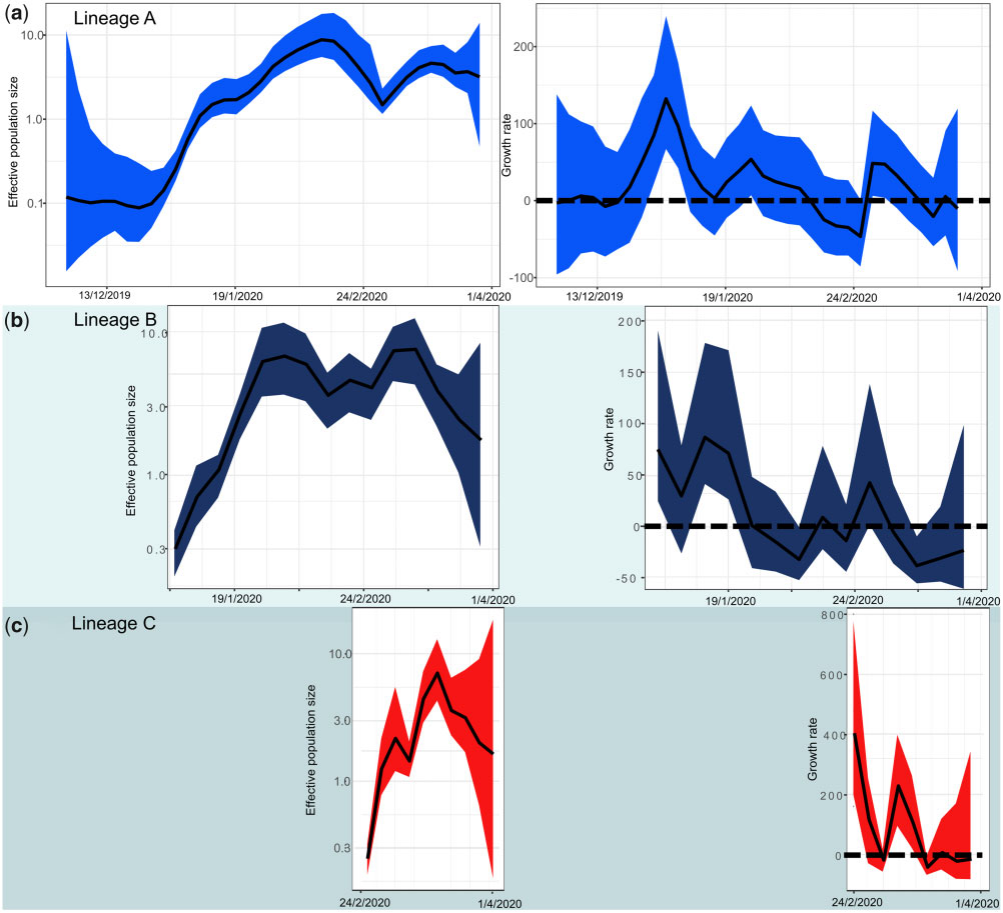
Effective population size (left panels) and growth rate of the effective population size per year (right panels) estimated through time for the three identified SARS-CoV-2 Lineages from our *skygrowth* models. The coloured 95 percent high probability density (HPD) intervals reflects lineages identified in Fig. 1. Dashed lines in the left panels indicate a growth rate of zero.

**Figure 3.**
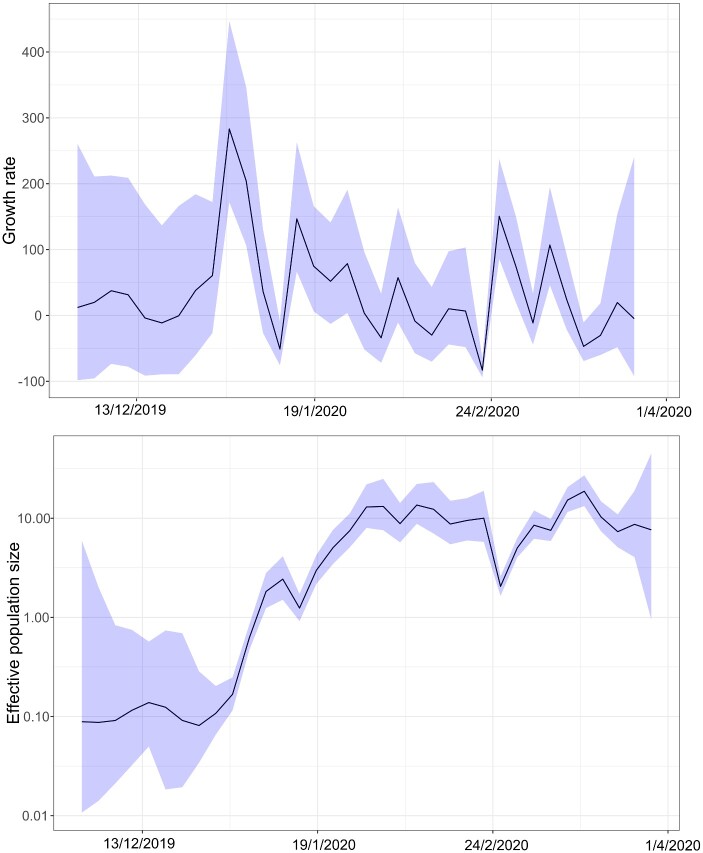
Growth rate (a) and effective population size (b) estimates through time from our *skygrowth* model using the complete dataset (all lineages of SARS-CoV-2). Light blue shading represents the 95% HPD of the estimates.

Furthermore, we show that these lineages have different demographic trajectories. Based on our maximum likelihood and Bayesian MCC time-scaled phylogenies, we estimated that Lineage A (and SARS-CoV-2 overall) diverged from its most recent common ancestor (MRCA) in November 2019 (95% high posterior density/confidence intervals November to December 2019, Fig. 1). Estimates from both approaches are comparable to other studies that have analyzed greater numbers of sequences (van Dorp et al. 2020). We also found support for rate variation across the phylogeny (coefficient of variation of rates: 0.12), although differences in MRCA estimates was minimal with strict and relaxed clock model having mostly overlapping distributions. Since emerging in China, our demographic analysis (Volz and Didelot 2018) suggests that the growth rate of the effective population size of Lineage A increased in early January (Fig. 2a), then decreasing throughout February before increasing once more. This dip coincides with control of the pathogen in China (Leung et al. 2020) and subsequent uncontrolled spread in Europe and North America. We found a similar pattern when we analyzed the complete dataset (Fig. 3a). The majority of sequences belonging to Lineage A originated from China in January to early February, whereas sequences from the USA, and Washington state, in particular, make up the majority of the sequences collated in March.

Our results support other analyses suggesting that Lineage B was derived from Lineage A and was not an independent introduction, even though Lineage B contains the earliest available genomes (Rambaut et al. 2020; Tang et al. 2020). Linked mutations in ORF1ab (8782, synonymous) and ORF8 (28144, non-synonymous) help to separate these lineages (as by Rambaut et al. 2020; Tang et al. 2020). Non-synonymous mutations in ORF14 (28881-3) also partially define these lineages, yet there is no evidence of phenotypic differences between these lineages. Further, there is a high degree of phylogenetic uncertainty about the node representing their most recent common ancestor and this lineage may be polyphyletic (Fig. 1, Supplementary Fig. S1). The growth rates of both lineages (Fig. 2) are also similar suggesting that the lineages were co-circulating, but more local investigation is needed to determine relative fitness differences. Soon after diverging from Lineage A, the growth rate of Lineage B was at its highest but then formed a pattern of peaks and troughs with the credible interval including zero (representing no growth) from January onward (Fig. 2b). The peak growth rate coincided with that of Lineage A (Fig. 2) indicating that this first wave of SARS-CoV-2 through China generated a relatively large amount of the genetic diversity. As many sequences classified in Lineage B originate from China (Fig. 1), the subsequent decline of this lineage may also be linked to control of the virus. There is also evidence for a rapid increase in growth rate of both Lineage A and Lineage B when spread increased outside of China and this coincides with the divergence date for Lineage C (Fig. 2).While our results on their own cannot rule out the possibility that the phylogenetic structure we identified was a result of founder effects (Korber et al. 2020) (i.e. the lineages diverged as they were transmitted to new locations), we used an eco-phylogenetic approach (Fountain-Jones et al. 2018) to quantify the geographic structure. We found that the sequences were not strongly clustered by country or continent (phylogenetic signal K < 0.15, Supplementary Table S2 see Section 2). However, for the continent contrast (i.e. modelling continent of origin for each sequence as a trait), this low K value was just significant using phylogenetically independent tip randomizations (*P* = 0.012, *Z* = −1.513, Supplementary Table S2). This is likely due to the large numbers of sequences from Lineage C from Europe.

Lineage C was predominantly European with no evidence that it circulated in China (Fig. 1). This lineage was well supported as monophyletic (node posterior support = 0.99, 91% bootstrap support, Fig. 1) and diverged from Lineage B in late January (95% highest posterior density late January to early February). Linked non-synonymous mutations differentiated this lineage in the S gene (sites 23402-04 or D614G) and ORF1ab (14407-09) regions. There is increasing evidence that the mutations in the S gene have resulted in phenotypic change in the virus [and the resultant changes to the Spike (S) protein] that has enabled this lineage more readily transmissible (Korber et al. 2020; The COVID-19 Genomics UK Consortium 2020). The mutations in the ORF1ab gene alter the RNA-dependent RNA polymerases (RdRp) that are crucial for the replication of RNA from the RNA template. There is evidence that this RdRp mutation may increase the mutation rate of the virus overall by reducing copy fidelity (Pachetti et al. 2020). The growth rate of Lineage C was initially high in late February, prior to the rapid increase of cases in Europe, but then declined, with one further peak around February 27, although the short duration suggests this may not be significant and could represent sampling noise. Accordingly, the effective population size of Lineage C increased rapidly during February to March, whereas there was only a small increase estimated for Lineage A and a decline in Lineage B (Fig. 2). Real-time phylogenetic reconstruction in Nextstrain (Hadfield et al. 2018), as well as results from intensively sampled populations in the UK (The COVID-19 Genomics UK Consortium 2020), have subsequently shown that this lineage has further expanded and is the most frequently sampled across the globe.

### 1.2 The growth and decline of SARS-CoV-2 lineages

We were able to identify three lineages that were not only genetically distinctive but also had unique demographic signatures, revealing insights into the underlying epidemiology of this pandemic. There is also increasing evidence that Lineage C is more transmissible than the other lineages (Korber et al. 2020; The COVID-19 Genomics UK Consortium 2020), revealing that our approach can detect important phenotypic changes to the virus. The number of cases increases day-by-day, as does the effective population size of the virus overall (Fig. 3b); both to be expected by their linear relationship in the early phase of a susceptible-infected-removed (SIR) compartmental model (Volz 2012). It appears that this increase is not distributed evenly across the phylogeny, with all lineages showing some evidence of decline at different times. However, there is bias in countries represented in the GISAID dataset we accessed, with, for example, no sequences in our dataset from the Middle East even though there was a significant (and ongoing) outbreak in this region. Further, our approach to identify non-random coalescent patterns does not account for phylogenetic uncertainty and future work is needed to address this limitation. Even though the outbreak is only months old at the time of writing, there is already sufficient genetic diversity to track the demographic trajectories of each lineage. Approaches such as the one presented here, combined with workflows quantifying geographical lineage dispersal (Dellicour et al. 2020), will be even more useful in the coming months to assess the longer-term impacts on SARS-CoV-2 control measures across the globe.

## 2. Methods

We downloaded 779 complete ‘high coverage only’ SARS-CoV-2 genome sequences from GISAID (Global Initiative on Sharing All Influenza Data; https://www.gisaid.org/, see Supplementary Appendix S1 for the acknowledgment information) (Elbe and Buckland-Merrett 2017) on the 24 March 2020. We aligned these sequences with MAFFT (Katoh and Standley 2013) using the CIPRES (Miller, Pfeiffer, and Schwartz, 2010) server and visually checked the results. We trimmed the first 130 bp and last 50 bp of the aligned sequences to remove potential sequencing artefacts in line with Nextstrain protocol (Hadfield et al. 2018). We tested for recombination in our alignment using RDP4 (Martin et al. 2015). We removed all duplicate sequences and sequences with more than 10 per cent missing data. We then constructed a Maximum Likelihood tree using IQ tree with 1000 ultrafast bootstraps (Nguyen, 2015) using the inbuilt model selection algorithm (‘ModelFinder’; Kalyaanamoorthy et al. 2017). We confirmed that there was a significant temporal signal in the dataset using root to tip regressions in TempEst (Rambaut, et al. 2016) (*R*^2^ = 0.19, correlation coefficient = 0.42). We removed sequences from Washington State and China that likely had some sequence error as they were strong outliers in the TempEst analysis. Removing sequence error, identical sequences and sequences with missing data reduced the dataset to 587 complete SARS-CoV-2 genomes.

We used both the maximum likelihood-based *treedater* method (Volz and Frost 2017) and a Bayesian approach to reconstruct the timing and spread of SARS-CoV-2. We employed the computationally intensive Bayesian methodology [BEAST version 1.10.4 (Suchard et al. 2018) with BEAGLE (Ayres et al. 2019) computational enhancement] to validate our maximum likelihood MRCA estimates and to provide dating estimates for internal nodes of interest. For the BEAST analysis, as there is strong evidence that the pandemic is growing, we assumed an exponential growth coalescent model. To estimate evolutionary rate, we compared runs using a strict and relaxed molecular clock. While we found some minor rate variation and very similar MRCA estimates, our ML results supported a relaxed clock model (see below), so subsequently we present results from that model. We performed each BEAST analysis in duplicate and ran the MCMC chains for 200 million iterations sampling every 20 000 steps. We visualized these results using Tracer (Rambaut et al. 2018) and ensured that all parameter estimates had converged with an effective sample size (ESS) > 200. We generated an MCC tree using *TreeAnnotator*, discarding 20 per cent as burn-in.

Our previously described ML tree was used as input of the *treedater* method (Volz and Frost 2017) to produce an ML time-scaled phylogeny. *Treedater* is an efficient maximum likelihood method that implements both a strict clock model using a Poisson process and a relaxed clock model using a Gamma-Poisson mixture. We compared the fit of relaxed and strict clock models using a parametric bootstrap test to compare the coefficient of variation of rates (Volz and Frost 2017) and used the best fitting model to construct the phylogeny as well. We estimated the confidence intervals for the dates of ancestors in this tree using parametric bootstraps.

We then used this time-stamped ML tree to test for structure within the tree using the non-parametric *treestructure* approach (Volz et al. 2020). Briefly, this method partitions the tips and internal nodes of a tree into discrete sets characterized by comparable coalescent patterns. See Volz et al. (2020) for analytical details. Given the relatively low levels of genetic diversity, we constrained our structure analysis to be able to identify a maximum of four lineages by making the minimum clade size 145 sequences and performed 100 000 tree simulations (with a significance threshold of 0.05). We then tested the hypothesis that each pair of identified clades within a tree were generated by the same coalescent process using the *treestructure* rank-sum test. We also performed the same analysis on the Bayesian MCC tree as well as 1000 trees from the posterior. To test if the identified lineages were a product of the founder effect, we modelled the geographic origin for each sequence as a trait across our phylogeny and measured the phylogenetic signal (the K statistic, Blomberg, Garland, and Ives 2003) of each trait using phylogenetic independent contrasts using the R package *Picante* (Kembel et al. 2010). We calculated K for both country of origin and continent of origin, and we tested the significance of K using 9999 randomizations. *K* = 0 represents little phylogenetic clustering by country or continent whereas *K* = 1 represents strong phylogenetic clustering.

For the complete dataset and each lineage subset, we modelled the effective population size growth rate through time using the *skygrowth* package (Volz and Didelot 2018). *Skygrowth* is a non-parametric Bayesian approach that applies a first-order autoregressive stochastic process on the growth rate of the effective population size. We parameterized our *skygrowth* models assuming that SARS-CoV-2 effective population size could change every three days. We used an exponential distribution with a mean of 0.1 to estimate the precision parameter (Τau). We ran the MCMC for 20 million generations thinning every 1000th sample and considered each analysis to be converged if the ESS >200. We compared our *skygrowth* models to *Skygrid* models using the R package ‘phylodyn’ (Karcher et al. 2017) using the default settings.

## Data availability statement

The ML tree and code used to perform these analyses are available here: https://github.com/nfj1380/covid19_evolution. BEAST log files are available upon request. 

## Supplementary data

Supplementary data are available at *Virus Evolution* online.

## Supplementary Material

veaa082_Supplementary_Data
