# Supplementary material for: Emerging phylogenetic structure of the SARS-CoV-2 pandemic
Source: Virus Evol. 2020 Nov 10;6(2):veaa082. doi: 10.1093/ve/veaa082 (PMC7717445; doi:10.1093/ve/veaa082)
Supplement: veaa082_Supplementary_Data [file veaa082_supplementary_data.zip › Supplementary material.docx]

**Supplementary material**


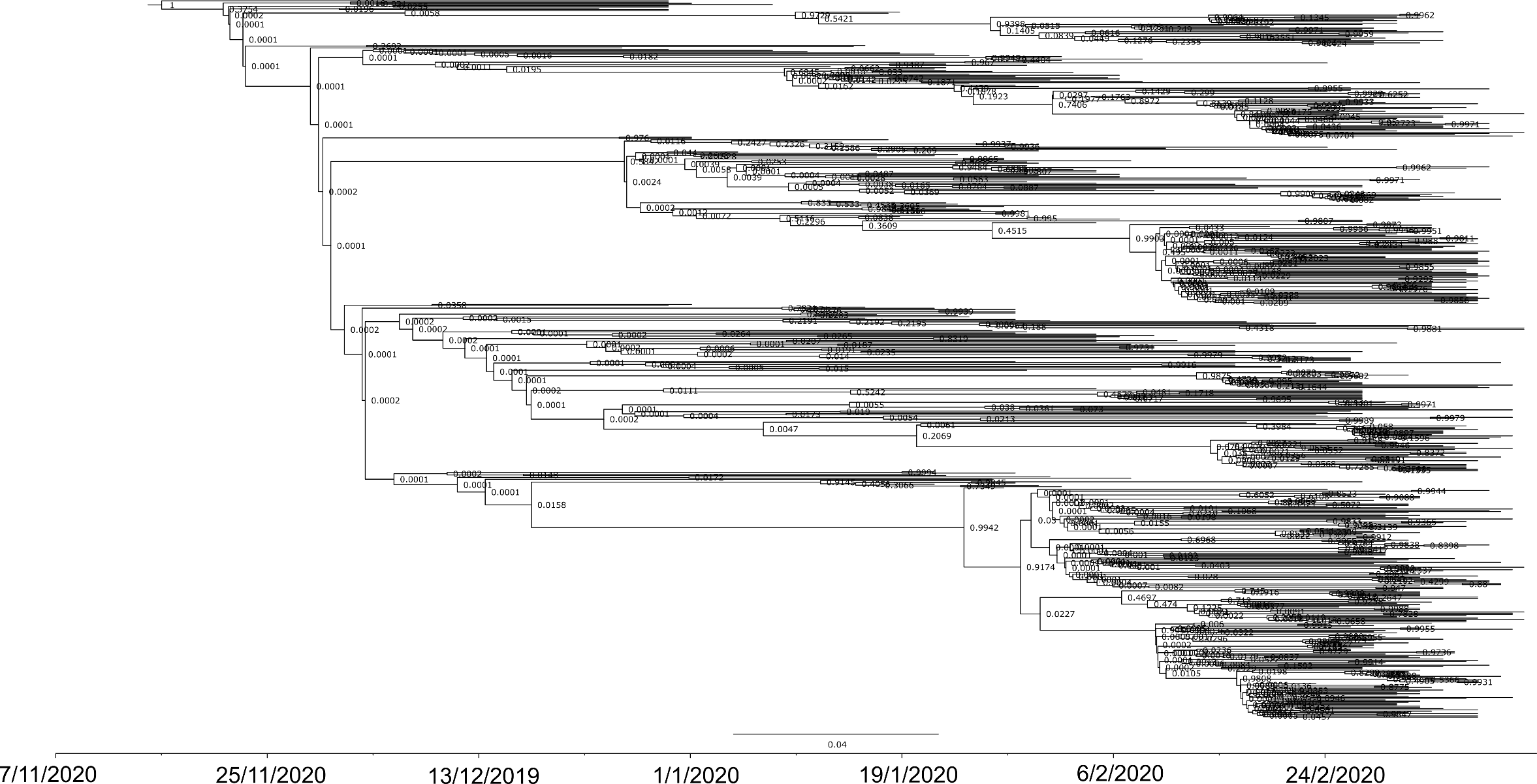


**Fig. S1.** Time-scaled Bayesian phylogeny showing all posterior support values.

**Table S1:** Results from *treestructure* tests on posterior trees from our Bayesian phylogenetic analysis. These results are based on1000 posterior sampled from our posterior tree set (see *Methods* for details)

| No phylogenetic structure | 2 significant clusters | 3 significant clusters |
| --- | --- | --- |
| 298 | 558 | 144 |

**Table S2:** Phylogenetic signal results modeling country and continent of origin as a trait on our *treedater* phylogeny based on K values. P-values are calculated based on variance of phylogenetically independent contrasts relative to tip shuffling randomization.

| **Trait** | K | Z | P |
| --- | --- | --- | --- |
| Country | 0.038 | -0.911 | 0.200 |
| Continent | 0.124 | -1.513 | 0.012 |


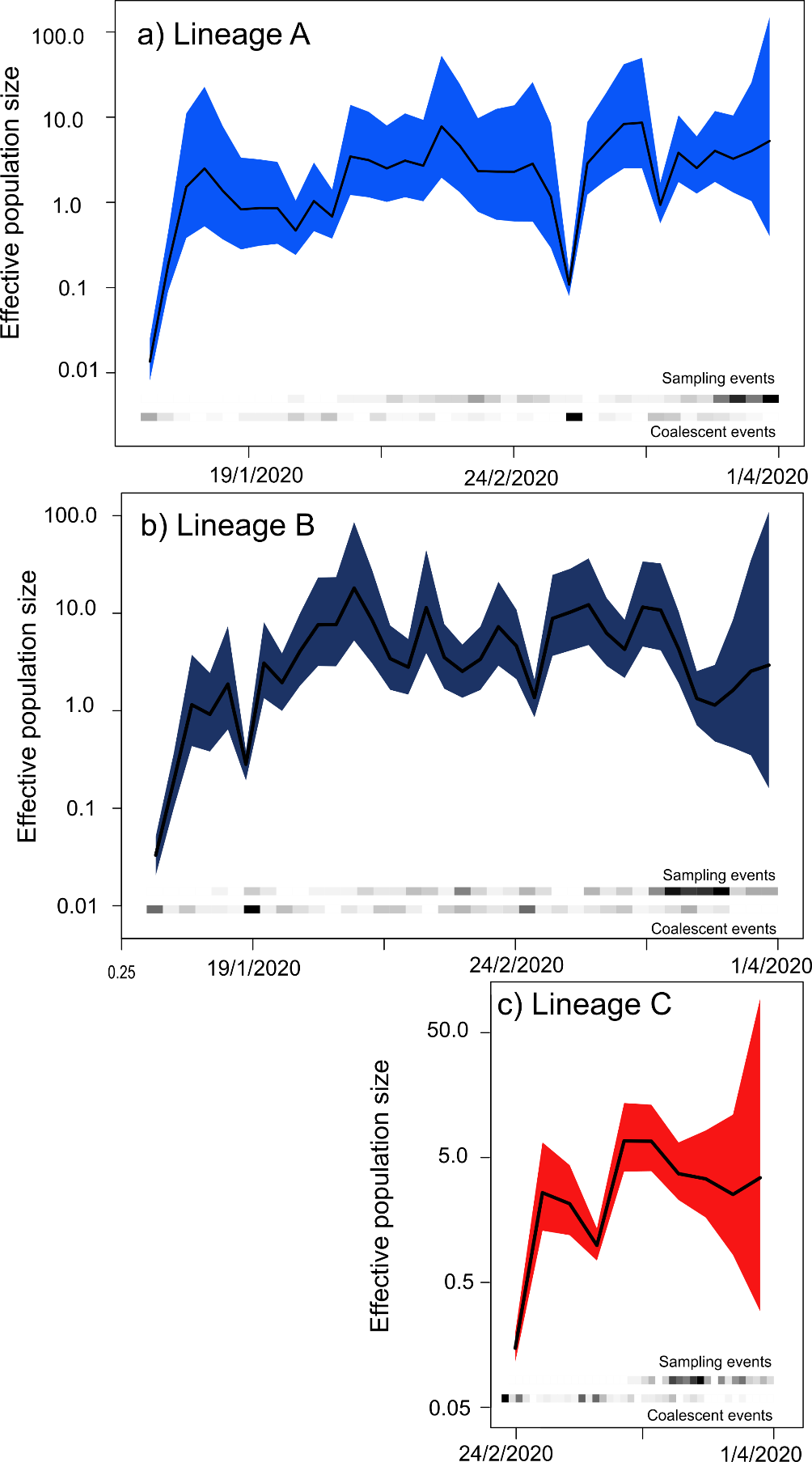


**Fig. S2.** Skygrid plots of the three identified SARS-CoV-2 lineages showing effective population size through time using the *phylodyn* approach (Karcher et al, 2017). The coloured 95% high probability density (HPD) intervals reflect lineages identified in Fig. 1.

**References**

1. Karcher, M. D., Palacios, J. A., Lan, S. & Minin, V. N. phylodyn: an R package for phylodynamic simulation and inference. *Mol. Ecol. Resour.* **17**, 96–100 (2017).
